# Perforated Appendicitis in a 23-Month-Old Child

**DOI:** 10.7759/cureus.82200

**Published:** 2025-04-13

**Authors:** Julián Aguilar, Alyssa Goldman, Adi Cohen, David Smith

**Affiliations:** 1 Emergency Medicine, HCA Florida Northwest Hospital, Margate, USA; 2 Graduate Medical Education, Nova Southeastern University Dr. Kiran C. Patel College of Osteopathic Medicine, Davie, USA; 3 Pediatric Emergency Medicine, HCA Florida Palms West Hospital, Loxahatchee, USA

**Keywords:** appendectomy, appendicolith, complicated appendicitis, inflamed appendix, pediatric surgery, perforated appendicitis

## Abstract

Appendicitis is caused by a blockage of the appendix, leading to the proliferation of intestinal bacteria and subsequent inflammation of the appendix. Within the scope of pediatrics, appendicitis is associated with an atypical presentation, and its symptoms can overlap with other abdominal conditions, contributing to a higher risk of misdiagnosis. Despite its relatively low prevalence in patients less than four years of age, appendicitis remains a critical condition that requires prompt recognition and intervention to avoid complications and ensure favorable outcomes. We present a case of perforated appendicitis in a 23-month-old female patient who presented to the emergency department with abdominal pain. This report highlights the challenges in diagnosing appendicitis in this age group and reviews current guidelines for the evaluation of suspected appendicitis in young children. Emphasis is placed on the importance of timely diagnosis and surgical intervention to reduce complications and prevent mortality.

## Introduction

While appendicitis is the leading cause of emergent abdominal surgery in childhood, it is a rare diagnosis in children less than four years of age, with an incidence of 0.01-0.06% [1]. Obstruction via mucus, stool, and infections leads to the inflammation of the appendix and disruption of intestinal blood flow [2]. Such infections can include viruses and parasites that cause lymph nodes in the intestine to enlarge, serving as a point of blockage [3]. In any case, the appendix swells, promoting the proliferation of bacteria [3]. The appendiceal walls lose their integrity due to stasis of blood flow, permitting the passage of foreign material into the abdomen and subsequent rupture of the appendix [2]. The risk of perforation is greatest among this age group, occurring at a rate of 51-100% of cases [1]. One explanation can be due to the underdevelopment of the omentum, which functions to regulate the immune system in response to inflammation, producing a fibrin seal to the affected area and clearing any bacteria present [4]. Nonspecific symptom presentation can also lead to a delay in diagnosis and, thus, surgical intervention [1]. Historically, appendicitis presents as periumbilical pain that migrates to the right lower quadrant [5]. The positioning of the appendix may not be as anticipated in children as a result of congenital abnormalities such as malrotation, situs inversus totalis, or a repaired hernia [1]. An appendicitis diagnosis made more than 36 to 48 hours after symptom onset is correlated to a perforation risk greater than 65% [5]. A perforated appendix subsequently leads to a 15-30% increase in appendectomy complications, including infection, abscess formation, intestinal obstruction, and adhesions [5].

## Case presentation

We present a case of a 23-month-old unvaccinated female child who presented to the emergency department with a two-day history of abdominal pain, fever, non-bloody emesis, and diarrhea. On primary examination, the patient was inconsolable and in significant distress secondary to abdominal pain. The abdomen was soft with guarding in the right lower quadrant; no masses were felt on exam, and the patient did not present with an acute abdomen. The patient was afebrile with stable vital signs, outside of mild tachycardia, and denied additional symptoms. Clinically, the patient appeared stable but in significant pain. The initial CBC demonstrated neutrophilic leukocytosis, while the CMP (comprehensive metabolic panel) demonstrated elevated C-reactive protein and total bilirubin levels (Table 1).

**Table 1:** Initial complete blood count results WBC: white blood cells; CRP: C-reactive protein

Parameter	Results	Reference Range
Segmented neutrophils (×10^3^ cells/µL)	71.7	1.5–7
WBC (×10^3^ cells/µL)	20.9	6.5–13
CRP (mg/dL)	4.6	<1.0
Total bilirubin (mg/dL)	2.2	0.1–1.2

**Figure 1:**
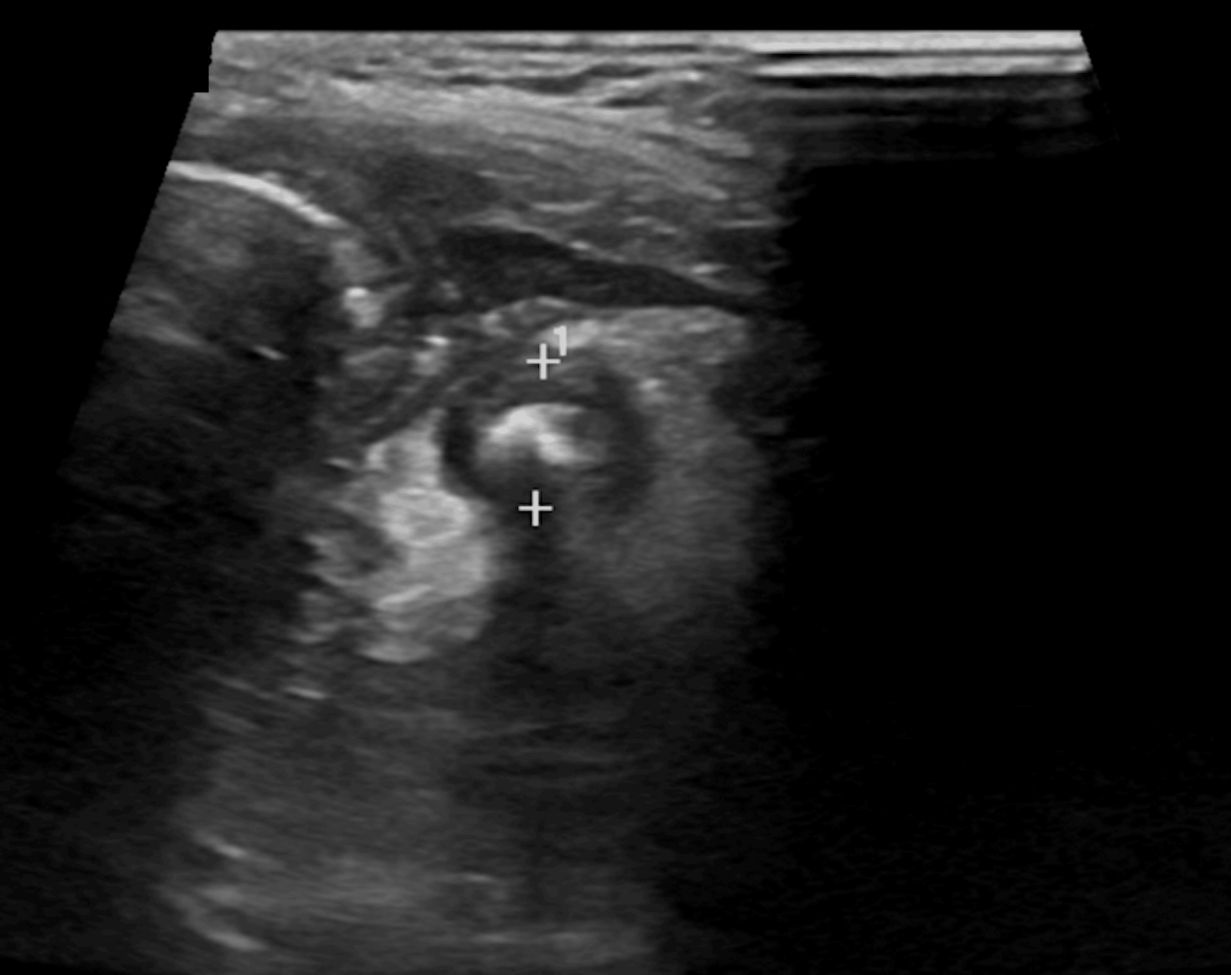
Abdominal ultrasound of the right lower quadrant demonstrating calcifications likely representing an appendicolith.

After administration of ondansetron for nausea and morphine for pain, the pain mildly subsided. With consideration for the clinical presentation, the differential diagnosis included gastroenteritis, intussusception, midgut volvulus, mesenteric adenitis, and appendicitis. An ultrasound of the abdomen demonstrated a hyperemic 7.5 mm blind-ending structure in the right lower quadrant of the abdomen with two associated shadowing calcifications likely representing appendicoliths, concerning for appendicitis (Figure 1). No significant free fluid was visualized within the abdomen. An apparent small bowel intussusception was seen that appeared to be resolved during the examination, as per the technologist. General surgery was consulted for further management.

**Figure 2:**
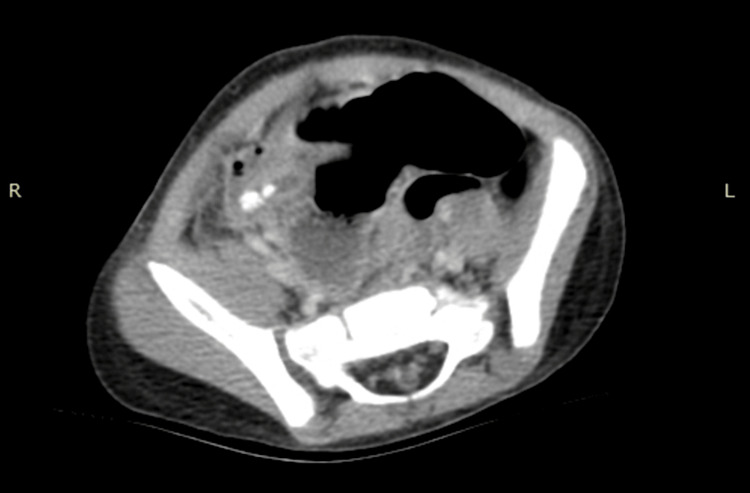
CT abdomen and pelvis demonstrating a dilated appendix with surrounding inflammation and trace fluid.

CT abdomen and pelvis was advised by the surgical team for diagnostic confirmation, which demonstrated a dilated appendix with at least two appendicoliths, surrounding inflammation with trace fluid in the right lower quadrant, and a phlegmon. There was no free air or abscess present (Figure 2). The patient was given a prophylactic dose of piperacillin and tazobactam with morphine for pain control and maintenance IVF (intravenous fluids) prior to surgery.

Upon admission, the patient was initially treated with supportive care and taken to the operating room, where she underwent an exploratory laparotomy for acute appendicitis. During surgery, it was discovered that the patient had ruptured appendicitis, and the general surgeon recommended admission to the pediatric intensive care unit (PICU) post-operatively. While in the PICU, the patient continued to improve with acetaminophen and morphine for pain control, fluids for hydration, and piperacillin and tazobactam for antimicrobial coverage. The patient was downgraded to the pediatric floor for continued management after tolerating a clear liquid diet. On the pediatric floor, the patient improved with multimodal pain control and continuation of antibiotics. Follow-up was scheduled for one week after hospital discharge to evaluate for post-operative complications, such as surgical site infection, and confirm completion of the antibiotics.

## Discussion

This case illustrates the need for a prompt diagnosis of appendicitis to ensure favorable patient outcomes. Our patient had an unremarkable past medical history. In the emergency department, an ultrasound was utilized to rule out possible etiologies of her presentation without radiation exposure.

The Alvarado score and Pediatric Appendicitis Score (PAS) are two systems that utilize clinical features and laboratory parameters to predict the likelihood of appendicitis [6]. These scoring systems aim to reduce radiation exposure and emergency department length of stay in certain children with abdominal pain [7]. However, it is stated that the accuracy of the Alvarado score is higher in children above the age of 10, and the PAS is only to be utilized in children 3-18 years of age [6]. Due to the lack of specificity for these scoring systems, clinician suspicion remains a mainstay for choosing to order an ultrasound.

Ultrasound remains the principal testing modality for appendicitis in children, as it is readily available and free of ionizing radiation exposure [8]. Sonographic findings indicative of appendicitis include visualization of a non-compressible appendix and a diameter greater or equal to 7 mm [8]. In a prospective, 10-center observational study by Mittal et al., ultrasound sensitivity and specificity for diagnosing children with appendicitis was found to be 72.5% and 97%, respectively. However, it was found that if the appendix was clearly visualized on ultrasound, the sensitivity significantly increased to 97.9% [7].

With high mortality rates and a difficult diagnosis, studies published in the Journal of Pediatric Surgery state that other diagnostic modalities should be considered if the appendix is not definitively visualized by ultrasound [9]. Despite meeting diagnostic criteria via ultrasound and clinically having a diagnosis of appendicitis, the choice to pursue a CT scan was chosen by the general surgery team due to the low incidence in this age group. CT has an exceedingly high sensitivity and specificity to confirm the clinical suspicion of appendicitis. Not only does it cause the patient less pain, but CT has much less operator dependence [10].

With an incidence of less than 1% in children under the age of four, a prompt diagnosis and treatment of appendicitis is challenging. Nonetheless, it should be considered in cases where a child presents with abdominal pain, vomiting, and extreme tenderness over the right lower quadrant. Ultrasound continues to be the primary diagnostic tool. When ultrasound is inconclusive, CT should be considered, as the risks of radiation exposure are outweighed by a missed diagnosis.

## Conclusions

This case underscores the critical importance of early recognition and intervention in managing perforated appendicitis in pediatrics. As evidenced by our patient's presentation with acute abdominal pain, fever, and diarrhea, this condition can often be portrayed clinically as nonspecific and difficult to diagnose. Physicians must maintain a broad differential diagnosis when faced with such symptoms to prevent a delay in diagnosis and medical intervention, which will diminish the risk of complications such as perforation. This case highlights the need for heightened clinical suspicion and reinforces the value of a multidisciplinary approach to optimize care and ensure better prognoses for children with perforated appendicitis.
